# On the nature of “skeletal” biofilm patterns, “hidden” heterogeneity and the role of bubbles to reveal them

**DOI:** 10.1038/s41522-019-0085-6

**Published:** 2019-03-21

**Authors:** Jesse Greener

**Affiliations:** 1 Faculté des sciences et de génie, Département de chimie, Université Laval, Québec City, QC G1V 0A6 Canada; 2 CHU de Quebec Research Centre, Laval University, 10 rue de l’Espinay, Quebec City, QC G1L 3L5 Canada

**Keywords:** Biofilms, Water microbiology

## Abstract

A short communication on the recent paper by Jang et al. discusses the role of “mushroom” structures and effects of nearly static bubbles on nascent biofilms.

## Correspondence

The effect of bubbles on surface-adhered microbiological systems in flow cells are usually associated with the applied wall shear forces during their transit, which can be significant. However, the role of nearly static bubbles on biofilm development is less studied. Therefore, we read with interest a recent paper by Jang et al. in your journal in which the authors studied the effects of slow moving bubbles across nascent biofilms of different ages within microchannels.^1^ Following their passage, beautiful semi-regular skeletal patterns were formed, depending on the biofilm age. In separate experiments, unperturbed nascent biofilms were stained with a lectin-based probe, which showed a “hidden heterogeneity” comprised of regions of accumulated extracellular polymeric substance (EPS) amongst otherwise continuous biofilm layers. The similarity to the skeletal patterns after bubble passage led the researchers to propose a mechanical “scraping” process under the bubble as a mechanism for bacterial rearrangement and accumulation at the relatively well-attached EPS sites, referred to as “bacterial levees”.

Not mentioned in the original paper is the striking resemblance of the observed features to the patterns formed by “mushroom-like” structures in many biofilms, including those of the *Pseudomonas aeruginosa* used by Jang et al. Mushroom structures also feature a hidden features, such as a network of open spaces surrounding localized anchor points at the attachment surface, which enhance nutrient exchange between the biofilm and the liquid phase. This, leads us to wonder if the structures observed by Jang et al. were not already there and that passage of the bubble simply revealed the underlying anchor points after shearing off the upper confluent biofilm layers. Here the question comes to confirmation of the initial 3D structure that was not visible from fluorescence images provided. Was it really planar as the authors suggested, or had the 3D structuration already started? Moreover, if it was truly a planar layer of bacteria containing hidden islands of EPS, as the authors propose, could these EPS structures have been the early-stage attachment points for future mushroom anchor points? This could inform previous dynamic studies by attenuated total reflection Fourier transform infrared spectroscopy (ATR-FTIR) that followed the development of 3D mushroom structures^2,3^ and the tendency for early formation of EPS chemical groups, such as polysaccharides.^4^ As well, it matches studies recently conducted by our group on *Pseudomonas sp*. biofilms using electrochemistry and time-lapse confocal laser scanning confocal microscopy (CLSM) in microchannels.^5^ In the latter case, CLSM images show a self-directed transition to similar skeletal structures shown by showed by Jang et al. under normal flow conditions—no bubbles required.

This is not to say that we doubt that static bubbles can affect biofilm properties. Quite the opposite. Recently, we also confirmed that surface-adhered bubbles can indeed have stark effects on nascent biofilms.^6^ Using CLSM imaging under a static bubble attached to a microchannel surface, we saw many small liquid pools, likely resulting from the rupture of a thin film as discussed by Jang et al. In the case that the bubbles formed on surfaces inoculated less than 30 mins earlier, we found that the pools were scattered throughout the field of view, with some containing bacteria at random. However, if similar measurements were made under bubbles which had been inoculated more than 2 h before, many of these liquid pools contained EPS and bacteria, which could protrude up to 10 µm from the surface. In all cases, the dry segments between the pools contained no observable bacteria. This supports the idea put forward by Jang et al. that loosely bound bacteria had been swept away under the force of the moving triple line from retreating liquid films until they became accumulated in EPS-rich locations. Were we seeing the same bacterial levees as reported by Jang et al, a few months earlier? Possibly. But the most interesting result in our studies was what happened after the bubble left the surface, which included pronounced effects on biofilm growth rates, homogeneity and even patterning. Therefore, we think it would be equally interesting for the authors to monitor the fate of the skeletal biofilms after the passage of the bubble.

Globally, we are hopeful that the work of Jang et al. can lead to more attention being paid to the effects of bubbles on biofilms. Moreover, their work is yet another magnificent example among a growing list showing how microfluidics can reignite fundamental research into bacterial biofilms.^7^
